# Antibiotic resistance and phylogenetic profiling of *Escherichia coli* from dairy farm soils; organic versus conventional systems

**DOI:** 10.1016/j.crmicr.2021.100088

**Published:** 2021-12-10

**Authors:** Omega Y Amoafo, Vanita Malekar, Eirian Jones, Stephen L W On

**Affiliations:** a Faculty of Agriculture and Life Sciences, Lincoln University, Springs Road Lincoln, Canterbury 7647, New Zealand; b Oceania Dairies Ltd., Morven 7980, New Zealand

**Keywords:** Antimicrobial resistance, Agrochemical, *Escherichia coli*, Conventional, Organic

## Abstract

The prevalence and spread of antimicrobial resistance (AMR) as a result of the persistent use and/or abuse of antimicrobials is a key health problem for health authorities and governments worldwide. A study of contrasting farming systems such as organic versus conventional dairy farming may help to authenticate some factors that may contribute to the prevalence and spread of AMR in their soils. A case study was conducted in organic and conventional dairy farms in the South Canterbury region of New Zealand.

A total of 814 dairy farm soil *E. coli* (DfSEC) isolates recovered over two years were studied. Isolates were recovered from each of two farms practicing organic, and another two practicing conventional husbandries. The *E. coli* isolates were examined for their antimicrobial resistance (AMR) against cefoxitin, cefpodoxime, chloramphenicol, ciprofloxacin, gentamicin, meropenem, nalidixic acid, and tetracycline. Phylogenetic relationships were assessed using an established multiplex PCR method. The AMR results indicated 3.7% of the DfSEC isolates were resistant to at least one of the eight selected antimicrobials. Of the resistant isolates, DfSEC from the organic dairy farms showed a lower prevalence of resistance to the antimicrobials tested, compared to their counterparts from the conventional farms. Phylogenetic analysis placed the majority (73.7%) of isolates recovered in group B1, itself dominated by isolates of bovine origin. The tendency for higher rates of resistance among strains from conventional farming may be important for future decision-making around farming practices Current husbandry practices may contribute to the prevalence and spread of AMR in the industry.

## Highlights

• First known comparison of antimicrobial resistance traits in *E. coli* strains from new zealand farms practicing organic and conventional husbandry.
• Potential extended spectrum β-lactamase producing strains isolated from dairy farm environments.
• Organic dairy farms tended to harbour fewer resistant isolates than those recovered from conventionally farmed counterparts.
• Evidence for anthroponotic transmission of resistant strains of human origin to farm environments.
• Implications for the spread of antimicrobial resistance traits from farm environments discussed.

## Introduction

The public health and economic impacts of antimicrobial resistance (AMR) are already significant, with predicted increases of major global concern (Jasovský et al., 2016; Naylor et al., 2018; Tacconelli and Pezzani, 2019).

Natural antibiotics (Raaijmakers and Mazzola, 2012) and the unquantifiable hundreds of millions of tons of human-made antimicrobials (Davies and Davies, 2010) eventually end up in environments such as agricultural fields and water bodies as whole compounds or their metabolites (Angeles et al., 2020; Ghirardini et al., 2020; Serra-Compte et al., 2017; Yuan et al., 2019). This may explain the positive correlation between the increased use of an antimicrobial group and the development of resistance against it (Korpela et al., 2016; Megraud et al., 2013; Seppälä et al., 1995). Furthermore, resistance traits may be transmitted and shared between strains (Zhuge et al., 2019) or along clonal lines, as observed within the phylogenetic groups of *E. coli* (Tarr et al., 2018).

New Zealand has a major agricultural economy with dairy farming as one of the major contributors to its gross domestic product; in 2018–2019, its dairy industry contributed 3% of global dairy production (DairyNZ, 2020). Conventional dairy farming, as opposed to organic dairy farming, contributes a significant amount of agrochemicals and their metabolites directly or indirectly into their environments (Chobtang et al., 2017; Mandal et al., 2020; Oliver et al., 2020; Singh et al., 2020). In the dairy farming industry, the importance of *E. coli* varies from its use as an indicator organism for faecal contamination of milk and other dairy food products (Oliver et al., 2020; Salaheen et al., 2019), to an agent of diseases of cows, including mastitis (Cui et al., 2020; Edgell, 2020); Nobrega et al., 2020).

Dairy farm soil *E. coli* (DfSEC) cycle between the soil environment with its variable physicochemical conditions of temperature, ultraviolet (UV) and visible light, pH, salinity, and the homeostatic rumenointestinal system of livestock. This occurs as the stock ingests and defecates, and the microbes from the grass and faeces assimilate into the soil matrix (Buchkowski et al., 2019; Müller et al., 2011; Mutschlechner et al., 2018). This situation may intensify the conditions of stress faced by DfSEC strains, and increase the rate of adaptation by mutation or gene acquisition by soil bacteria to improve their survival (Lori et al., 2017; van Bruggen et al., 2019). The high-level dependence of conventional dairy farming on agrochemicals together with commercial antimicrobials compared to the limit/non-usage of commercial agrochemicals in organic dairy farming may also affect the microbes in their soils differently.

In this study, we compare the rates of AMR determined in 814 dairy farm soil *E. coli* isolates recovered over two years from dairy farms in Canterbury, New Zealand, that employed either conventional or organic farming approaches, to assess any differences such farming systems may have on the emergence of AMR in this ubiquitous and model organism.

## Materials and methods

### Sampling times and sites

Dairy farm soil samples were collected from two conventional dairy farms and two organic dairy farms located within a 25 km radius in the Southern Canterbury town of Geraldine in the South Island of New Zealand over 4 four sampling times (Table 1).

**Table 1:** Dairy farm soil sampling schedule.

Sampling order	Season	Month	dairy farm practice	year
first soil sampling	spring	October	start of calving to start of mating	2017
second soil sampling	spring	October	start of calving to start of mating	2018
third soil sampling	autumn	March	milking/grass pasture management	2018
fourth sampling	winter	June	milking/crop pasture management	2018

The Geraldine and Pleasant Point regions are a farming community involved in crops (grains and vegetables), forestry, and animal (dairy cattle, sheep, and deer) farming. The region comprises the counties of Geraldine, Levels, Mackenzie, and Waimate. It is bounded in the north by the Rangitata River, Forest Creek, and part of the Two Thumb mountain range, in the west by the crest of the Southern Alps, in the south by the Waitaki River, and in the east by the Pacific Ocean. Of the total area of 137 600 km^2^, 86% is farmed. The soils of this Canterbury region are silty sandy loams, formed mainly from greywacke alluvium. The soils have variation in-depth as they are underlined with gravel and boulders. The soil may be stony throughout its profile or maybe 20 to 100 mm of silt or sandy loams above the shingle (Landcare Research, Soil Map online https://smap.landcareresearch.co.nz/). The vegetation type on most dairy farm fields (paddocks in NZ) is ryegrass (*Lolium* sp.) with white and red clover (*Trifolium repens* and *Trifolium pratense*) swards. Two organic dairy farms, each of which has a conventional dairy farm within 5–10 km distance were chosen for this study. The Clearwaters (CW) organic dairy farm (GPS: 44°15′53.8″S 171°10′11.2″E) and Peel Forest (PF) conventional dairy farm (GPS: 44°00′50.4″S 171°16′26.4″E) are slightly closer together, about ⁓5 km apart. While the Totara Valley (TL) organic dairy farm (GPS: 44°14′17.5″S 171°04′02.1″E) and Mill Road (MRD) conventional dairy farm (GPS: 44°16′23.9″S 171°10′12.7″E) are also about 5 km apart. All four farms are located within a ⁓20–25 km radius.

### Sampling procedure

To ensure that the *E. coli* isolates obtained from the soil of the dairy farms have had interaction with the stock held on the farm, the following sampling procedure was adopted for the farms during all the sampling times indicated in Table 1. From each paddock, ⁓10 *E. coli* DfSEC isolates (10×5 × 4. Table 1) were selected for future study.

• The map of a farm was viewed and the paddock layout determined. Five paddocks that had been grazed within the last 24–48 h were chosen for soil sampling.
• Soil samples were taken from five selected spots on a paddock at ⁓200 g of soil per spot using a hand-held auger of 40 mm diameter and 300 mm depth.
• Spots were selected around the drinking trough on a paddock and from the paddock gate to the trough. Most of the stock on a paddock would have drunk from the trough and defecated in the area.
• Soil samples were taken at depths of between 50 and 3 000 mm from the surface depending on the presence or absence of boulders at a chosen spot.
• The ⁓200 g of soil from each of the 5 chosen spots were composited together in a sterile 1 000 ml Shott bottle to make ⁓1 kg of soil sample/ paddock/ sampling time.
• An aseptic technique was used to prevent contamination of samples between different farms. Equipment was washed with tap water, disinfected with 1% Virkon™ solution dried, and sprayed with 70% ethanol.
• All samples were processed within 24 h of their collection after taking to the laboratory for the isolation process.

### Isolation procedure

In the laboratory, 25 g of a paddock's composited soil was mixed with 225 mL of EC broth (Oxoid™ CM0853 Thermo Scientific™, Auckland, NZ) in a 2 000 mL sterile stomacher bag. The stomacher bag with the soil was then shaken using a stomacher (Interscience BagMixer®, France) for one min at three stroke/s, to form a soil slurry. The soil slurry was then put into a sterile cotton-plugged 500 mL conical flask and incubated in a shaker incubator (Thermo Scientific™ MaxQ 4 000, Auckland. NZ) at 44.5 °C to limit clonal multiplication (Hunke and Betton, 2003; Jozefczuk et al., 2010; Lenski and Bennett, 1993) while shaking at 1 g for 8–12 h (Hakalehto et al., 2010; Patel et al., 2014). One mL of solution was pipetted from the supernatant into 9 mL of sterile PBS solution in 15 mL Eppendorf® tubes and gently vortexed to represent a 10^−1^ dilution. A serial dilution was then prepared from 10^−1^ to the 10^−6^ diluent. From the diluents, 100 µL of the solution was plated onto MacConkey agar (Oxoid, CM0945 Thermo Scientific™, Auckland, NZ), spread, and incubated at the *E. coli* physiological optimum temperature of 37 °C for 24 h in duplicate (Kobayashi et al., 2007). For each sample processed, 50 colonies with typical *E. coli* characteristics of pink colouration with precipitate were selected from the MacConkey agar plates and subcultured, sequentially, onto EMB agar (Oxoid, CM0069 Thermo Scientific™, Auckland, NZ), and then NMUG agar (Oxoid, CM0978 Thermo Scientific™, Auckland, NZ), for assured phenotypic identification. A 0.5 nm OD_600_ (SmartSpec® Bio-Rad Laboratories Pty. Ltd, Auckland, NZ) cell suspension of *E. coli* ATCC25922 was prepared and plated on the selected media for comparison, as a positive control at each culturing step.

### Species identity confirmation by PCR

**Fig. 1:**
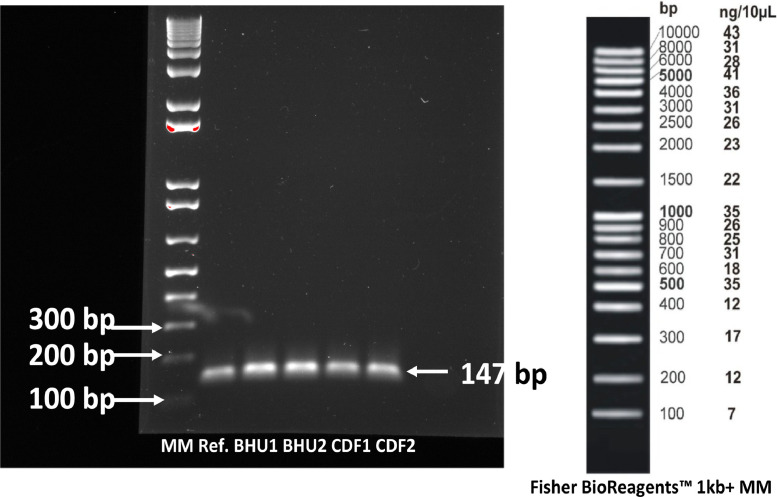
Examplar of PCR confirmation of dairy farm *E. coli* isolates using primers of Bej et al., 1991. MW, Molecular marker 1kb+ (Fisher BioReagents™, Thermo Scientific™ Auckland, NZ); Ref., Reference strain *E. coli* ATCC25922; BHU1, BHU2 = Organic Dairy Farm isolates 1&2; CDF1, CDF2 = Conventional Dairy Farm isolates 1&2.

Isolates were later cultured on TBX agar (Oxoid, CMO945) for identity confirmation by PCR according to Bej and Dicesare (Bej et al., 1991b). Briefly, the tip of a sterile 200 µl pipette tip was used to pick *E. coli* cells from a separated single colony and cells suspended in 20 µl ultrapure DNA/RNA-free water (GIBCO™, Thermo Scientific™, Auckland, NZ) bacterial cell in a 1.5 ml Eppendorf® tube. The cell suspension was then heat-lysed (Brian et al., 1992) at 95 °C for 5 min in a heat block (AcuBlock™ Labnet International INC. NJ, USA) and centrifuged (Eppendorf® Minispin® plus, Sigma-Aldrich, Auckland, NZ) at 4 000 g for 5 min; a 2 µL aliquot of the heat lysate was then used as the template for the *E. coli-*specific PCR (Bej et al., 1991a). All DfSEC isolates used for the study were confirmed by PCR. Primers used were: Forward; 5′-AAAACGGCAAGAAAAAGCAG-3′ and Reverse; 5′-AC GCGTGGTTACAGTCTTGCG-3′, located within the *uid*A structural gene of *E. coli* as outlined by Bej et al. (Bej et al., 1991b) and also cited by numerous authors (Brasher et al., 1998; Chigor et al., 2020; Iqbal et al., 1997; Khan et al., 2007; Kibbee et al., 2013; Maheux et al., 2009; Molina and Lowe, 2012; Ntuli et al., 2017). A 20 µL master mix of 0.2 µL of 2 U Taq polymerase, 2 µL of 10X PCR buffer, 2 µL of Q (Bio-Rad Laboratories Pty. Ltd, Auckland, NZ), 2 µL of 25 mM MgCL_2_, 0.8 µL each of forward and reverse primers (10 mM), 0.8 µL of dNTPs (10 mM) with DNA template and made up to 20 µl with ultra-pure DNA/RNA-free water. The mixture was placed into a thermocycler (Labnet MultiGene TC 9600 G. Sigma-Aldrich, Auckland NZ) for 30 cycles at 94 °C, denaturing for 1 min, and primer annealing at 55 °C for 1 min and extension at 72 °C for 3 min. The PCR product was visualised following a 2% agarose gel electrophoresis with 0.07 µL Sybrsafe (Invitrogen®, Auckland, NZ)/mL of gel, run at 90 V for 60 min and visualised with a molecular imager (Gel Doc™ XR+ Bio-Rad Laboratories Pty. Ltd, Auckland, NZ). The farm soil *E. coli* isolates were compared to *E. coli* ATCC25922 for the *E. coli* specific molecular band size of approximately 147 bp (Brasher et al., 1998) referenced to the 1 kb+ molecular marker (Fisher BioRFeagents™, Thermo Scientific™ Auckland, NZ) (Fig. 1).

### Antimicrobial susceptibility/resistance profiling of DfSEC

Antibiotic susceptibility testingwas conducted on 841 DfEC isolates collected from all four of the study farms over the four sampling times using the disc diffusion method described by the European Committee on Antimicrobial Susceptibility Testing (EUCAST, 2015) on eight selected antimicrobials (Table 2). The criteria for the selection of the group of antimicrobials (Table 2) for this study was based on the following rationale.

(a) cefpodoxime as a third-generation cephalosporin and ESBL (Shankar and Balasubramanium, 2014).
(b) chloramphenicol, ciprofloxacin, gentamicin, meropenem, and tetracycline as listed by the World Health Organisation (WHO) essential medicine (Organization, 2019).
(c) cefoxitin and meropenem as the yardstick for a potential ESBL resistant organism,as recommended by EUCAST for antimicrobial susceptibility testing (EUCAST, 2019).
(d) nalidixic acid as a synthetic antimicrobial, resistance to which may be as the result of human activity only (Kyzioł et al., 2020; Michael et al., 2014).

**Table 2:** List of antimicrobials and their concentrations used.

Antimicrobial	Concentration µg	Symbol
cefoxitin	30	FOX30
cefpodoxime	10	CPD10
chloramphenicol	30	C30
ciprofloxacin	30	CIP30
gentamicin	10	CN10
meropenem	10	Mem10
nalidixic acid	30	Na30
tetracycline	30	Te30

Briefly, Mueller-Hinton (MH) agar plates (Oxoid CM0337 ThermoScientific™, Auckland, NZ) were prepared according to the manufacturer's instructions. A solution of the agar base was sterilized by autoclaving at 121 °C for 15 min and poured into a sterile Petri dish to a depth of 4 mm, dried, and stored in plastic bags at 4 °C until use (Cockerill, 2011). Plates of MH agar used at all times were fresh, not more than five days old. A single colony of the dairy farm soil isolated *E. coli* growing on a TBX agar plate at 24 h was inoculated into 5 mL of 0.1 M PBS solution using a sterile microbiological loop and gently vortexed for 10 s. The turbidity of the bacteria suspension was compared to the turbidity of 0.5 McFarland standard solution (0.5 mL of 0.048 M BaCl_2_ to 99.5 mL of 0.18 M H_2_SO_4_) and measured by a spectrophotometer (SmartSpec3000™ Bio-Rad Laboratories Pty. Ltd, Auckland, NZ) to be between 0.08–0.1 nm OD_600_ (EUCAST, 2013).

A fresh sterile cotton bud was immersed in the bacterial suspension and pressed against the bottle container for 2–3 s to remove the excess bacterial suspension and then used to make an initial mat spread onto the MH agar plate. This was repeated after turning the plate at 90° to obtain a uniform spread of bacteria on the agar surface. Antimicrobial discs (Oxoid™ Thermo Scientific™, Auckland, NZ) for selected antimicrobials (Table 2) stored in a desiccant at 4 °C were placed on the agar and firmly pressed using forceps sterilised by dipping in 95% ethanol and flamed.

The agar plates were first held with the right-side-up for about 5 min and later inverted (within 15 min of plating) and incubated at 37 °C for 24 h (Andrews and Testing, 2001). The inhibition zone diameters (mm) at the point of inhibition were measured using a ruler and interpreted into resistant (R), intermediate (I) and susceptible (S) reactions according to the European Committee on Antimicrobial Susceptibility Testing EUCAST (2015) breakpoints for the disc diffusion method of antimicrobial susceptibility testing (Table A1.1). Inhibition zones of the dairy farm soil isolated *E. coli* were compared to reference *E. coli* NCTC12241 and *E. coli* ATCC25922 in all cases as recommended by the EUCAST (2015). Records of the antimicrobial profile of at least 50 PCR confirmed *E. coli* (Bej et al., 1991a) from each farm for each of the four sampling times (spring 2017, spring, autumn, and winter of 2018) were made in Microsoft Excel.

### Statistical analysis

Antibiotic resistance data were recorded in binary format (1 for resistant and intermediate results, and 0 for fully sensitive) for statistical analysis. To calculate the odds ratio between farms, any resistance trait exhibited by a strain was considered to denote resistance without further consideration of the number of antimicrobials strains were resistant to. To calculate P- values and odds ratios between seasons, every resistant trait examined for was considered in the analysis. Data were analyzed using the “fit binary regression model” in Minitab19 statistical software (MiniTab LLC, Pennsylvania, USA).

### Phenotypic screening of dfsec for ampc and ESBLs production

**Fig. 2:**
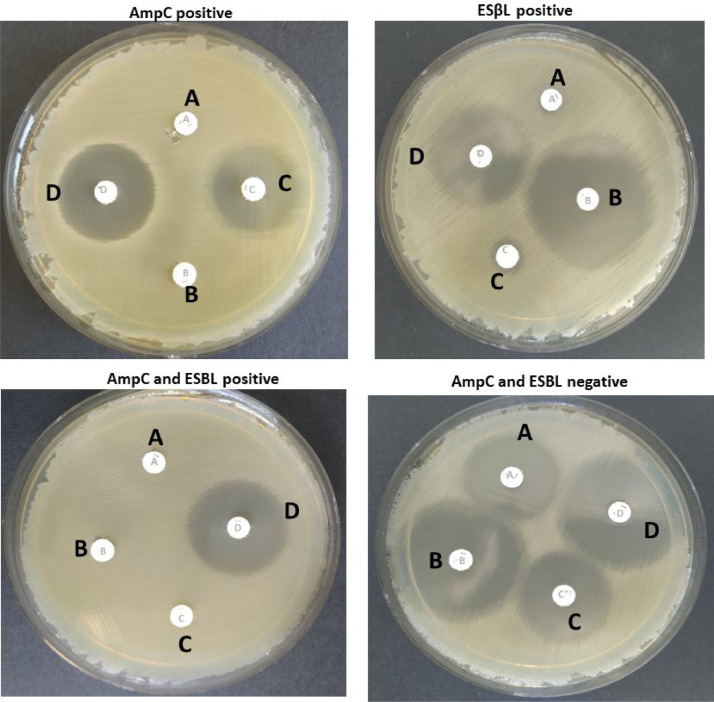
Exemplar of phenotypic screening for AmpC and ESBL-producing dairy farm soil *E. coli* isolates. For the detection of AmpC with porin loss KPC and MBL activity, the D73C (MAST™ Group Ltd, Liverpool, UK) the interpretations were as follows: B*–A* ≥ 5 mm; C*-A* and D*-A* 〈 5 mm → MBL activity. C*–A*, ≥5 mm; B*-A, D*-A*<5 mm → KPC activity. Distances between discs A*, B* C*, and D* ≤ 2 mm and *E* < 10 mm → OXA-48 positive. Distances between discs A*, B*, C*, and D* ≤ 2 mm and E 〉 10 mm → AmpC, KPC, OXA-48 negative.

For the detection of AmpC and/or ESBL enzyme-producing *E. coli* isolates, MASTDISCS® Combi AmpC and ESBL Detection Discs D68C commercial kit (MAST™ Group Ltd, Liverpool, UK) were used for the phenotype screening procedure according to the EUCAST (2015) protocol. Briefly, a single *E. coli* colony from a TBX agar plate not more than 24 h old was used. The cell concentration, method of spread, and disc placement were carried out similarly to the disc diffusion method of the antimicrobial susceptibility testing procedure described previously. All four discs (A, B, C, and D) were placed on an agar plate with sufficient spacing between them so as not to fuse inhibition zones. Weekly quality control of disc performance was conducted using a negative control *E. coli* ATCC25922 during the phenotype screening periods. The interpretation of the test results was made according to the manufacturer's instructions (cf. Fig. 2).

### Phylogenetic grouping of DfSEC

The 814 DfSEc isolates were grouped into groups A, B1, B2, C, D, E, or clade I, II, III, IV, or V phylogenetic of *E. coli* using primers and the protocol described by Clermont et al. (2013). The supernatant of heat-lysed DfSEC cells from a single colony cultured on a nutrient agar plate was inoculated into 20 µL of molecular grade water and 1 µL was used as a DNA template for the PCR reaction. Briefly, 20 µL PCR reaction was set up using 2 µL of 10X PCR buffer, 0.4 µL of 2 µM of each dNTP, 0.4 µL of 2 U Taq polymerase, 2 µL each of primers at 20 µM concentration of forward (f) and reverse (r) for *chu*A, *yj*A, *Trp*. For *ace*K (f) and *arp* (r), however, 2 µL of primers at 40 µM concentration and for TspE4C2.1b and TspE4C2.2b, 1 µL of each primer at 40 µM concentration were used per reaction. The reaction mixture was completed with 1.2 µL of 25 mM MgCL_2_, 2 µL of molecular grade distilled water, and 1 µL of the DNA template.

**Fig. 3:**
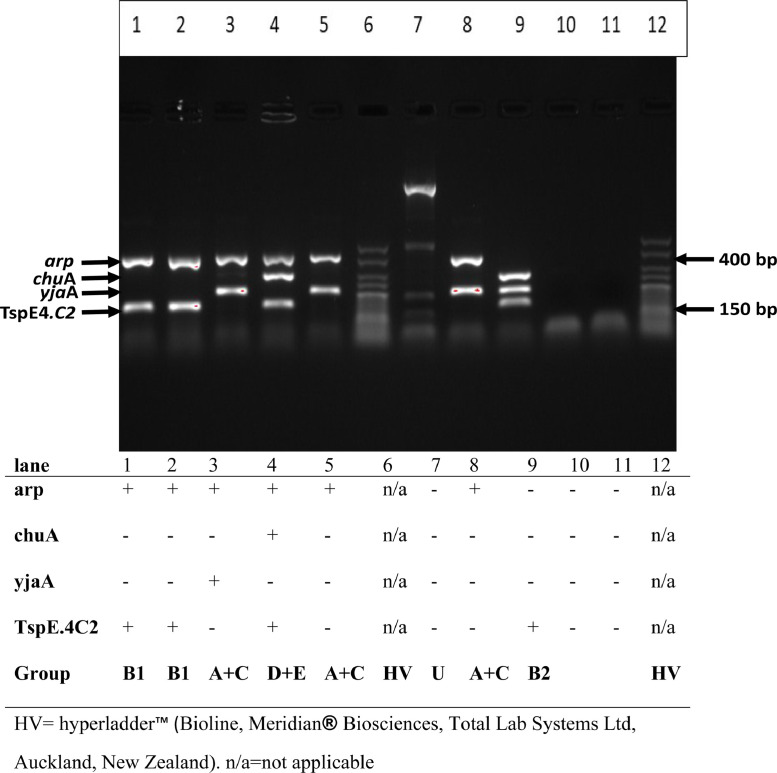
*E. coli* quadruplex phylogenetic group assignation. Based on (Clermont et al., 2013).

The reactions were set in a thermocycler (Labnet MultiGene TC 9600 G Sigma-Aldrich, Auckland, NZ) with the following protocol: denaturing for 4 min at 94 °C, 30 cycles of 5 s at 94 °C and annealing at 20 s at 57 °C for group E or 59 °C for the quadruplex and group A + C differentiating PCRs and final extension step was set for 5 min at 72 °C. The PCR primers used for the allele-specific phylogroups C and E were trpAgpC.f and trpAgpC.r at 12 µM concentration each and ArpAgpE.f and ArpAgpE.r, at 40 µM concentration each, respectively. The concentrations of dNTPs, 10X buffer, and MgCL_2_ remained as per the quadruplex PCR reaction and made up to 20 µL final volume with molecular grade water. The final product was stored at 4 °C until the PCR product was run on 2% agarose gel (2 g molecular grade agarose to 100 mL of 1 M tris EDTA buffer) electrophoresis using 0.07 µL Sybrsafe (Invitrogen, Auckland, NZ)/mL of gel, run at 90 V for 60 min, visualised and photographed with a molecular imager (Gel Doc™ XR+ Bio-Rad Laboratories Pty. Ltd, Auckland, NZ) (Fig. 3). Statistical analysis was done using t-tests or ANOVA, as appropriate, in SigmaPlot14.0 statistical software (Systat software, San Jose, USA).

In this study, the phylogenetic group assignation was done according to the guidelines of Clermont et al. (2013). Briefly, the presence (+) or absence () of a quadruplex PCR product band in a lane corresponding to an isolate was marked according to the molecular mass of the band. The phylogenetic group was assigned according to the final analyzes of the band's absence/presence (-/+) as outlined by Clermont et al. (2013) (Fig. 3), using the hyperladder™ V (HVL) (Bioline, Meridian® Biosciences, Total Lab Systems Ltd, Auckland, New Zealand). In cases where an isolate was initially typed either as A + C or *D* + *E* phylogenetic group, a second PCR was done using the primers *trp*AgpC.f, *trp*AgpC.f and ArpAgpE.f, ArpAgpE.r, respectively at 12 µM concentration (Lescat et al., 2013), with similar PCR components of buffer, 25 mM MgCL_2_, dNTP, DNA template, and molecular grade water into a final PCR reaction of 20 µL. The primers *trp*BA.f and *trp*BA.r also at 12 µM concentration were included as an internal control to help differentiate between phylogenetic group E and clade *I*+II (Clermont et al., 2013).

An isolate, when assigned a preliminary group of *D* + *E*, E+Clade *I*+II, or A + C was re-assigned its final phylogenetic group after confirmation or denial of the presence of a band when a secondary PCR with the trp*Agp*C or try*Agp*E primer in a duplex PCR reaction corresponding to a group C or E, respectively instead of a group A or D accordingly. Statistical analysis of the phylogenetically typed 814 *E. coli* isolates collected from the four farms over the four-time points was done using the Mann-Whitney Rank Sum *t*-test or by Kurskal-Wallis ANOVA on ranks, as appropriate.

## Results

### DfSEC isolation

For each sampling period, at least 50 dairy farm soil *E. coli* (DfSEC) isolates per farm whose identity was confirmed by PCR (Bej et al., 1991a) were isolated and stored in a 25% glycerol stock in brain heart infusion broth at a temperature of −80 °C for future analysis. In total, for this study, 814 DfSEC were isolated over the four-time sampling periods (Table S1).

### Antimicrobial resistance profiling of DfSEC

The antimicrobial resistance testing of DfSEC isolates from the four farms, over four sampling times against eight different antimicrobials with a total of 7 224 tests (Table S1) showed that 3.7% of the isolates tested were resistant to at least one of the eight selected antimicrobials. Statistical analysis showed a significant difference (*P* < 0.0001) between the eight different antimicrobials used and the sampling time point of the DfSEC isolates. When the different farming systems were compared, the conventional dairy farms were 1.18–1.89 times more likely to be resistant compared to the organic dairy farms (Table 3). By contrast, little difference between the number of resistant strains found in conventional farms was seen, but the Totara Valley organic farm had a higher chance of the DfSEC isolates being resistant compared to the Clearwaters organic farm with an odds ratio of 1.27 (Table 3).

**Table 3:** Comparison of AMR rates of DfSEC isolates from each organic and conventional dairy farm using the odds ratio metric.

Conventional farm		Organic farm	Odds ratio
Mill Road		Totara Valley	1.33
Mill Road	vs	Clearwaters	1.89
Peel Forest		Clearwaters	1.55
Peel Forest		Totara Valley	1.18
	**Conventional farms**		
Peel Forest	vs	Mill Road	0.82
	**Organic farms**		
Totara Valley	vs	Clearwaters	1.27

Combined data from the two organic farms were compared to that from the two conventional farms on a seasonal basis (Table 4). The spring of 2017 data showed a significant difference (*P* < 0.01), with a higher prevalence of resistance in the organic farms compared to the conventional farms (odds ratio 1.72). This data was skewed by 12 out of 50 isolates from the Clearwaters organic farm being resistant against tetracycline (Te30) at that sampling time. In the spring and winter of 2018, however, the prevalence of resistance was significantly higher (*P* < 0.0001) in the conventional farms compared to the organic farms (odds ratio of 0.20, 0.08), respectively. In the autumn of 2018, there was no significant difference between the two farming systems regarding the prevalence of resistance (*P* = 0.29) but the organic farms showed an odds ratio of 0.74 less chance of showing resistance compared to their conventional counterparts (Table 4). In this study, a comparison of the percentage resistance of the 814 DfSEC to the eight selected antimicrobials was compared between the organic farm soil isolates and their conventional farm counterparts at each sampling time point. The P-value and odds ratio of the comparison was assessed. Overall, DfSEC isolates from the organic dairy farms showed a lower prevalence of resistance to the antimicrobials tested, compared to their counterparts from the conventional farms. (Table 4).

**Table 4:** Percentage of DfSEC isolates exhibiting resistance against selected antimicrobials per season and per farming system. Odds ratios and P-values calculated as described elsewhere.

		% of DfSEC resistant to selected antimicrobials between conventional and organic dairy farms											
Sampling–time point	Farming system	FOX30	CPD10	CIP10		C30		CN10	Mem10	Na30	Te30	P-value	Odds ratio
Spring 2017	organic	36.9	9.6	0.0	0.0		0.0		12.3	0.0	11.4	<0.01	1.72
	conventional	21.0	5.4	0.0	0.0		2.0		26.9	2.9	6.7		
Spring 2018	organic	7.7	0.0	0.0	0.0		0.9		0.0	0.9	0.0	<0.0001	0.20
	conventional	12.9	0.9	0.0	3.9		0.0		12.0	0.9	7.2		
Autumn 2018	organic	15.4	4.8	0.0	2.9		3.8		2.0	0.0	1.9	0.29	0.74
	conventional	13.1	0.0	0.0	0.0		0.0		3.0	0.0	4.3	<0.0001	
Winter 2018	organic	5.1	0.0	0.0	1.0		0.0		2.0	0.0	0.0		0.08
	conventional	20.5	5.9	0.0	7.0		3.0		0.9	1.0	1.0		

FOX30 = cefoxitin 30 μg/disc: CPD10 = cefpodoxime 10 μg/disc: CIP10 = ciprofloxacin 10 μg/disc: C30 = chloramphenicol 30 μg/disc.

CN10 = gentamicin 10 μg/disc: Mem10 =  meropenem 10 μg/disc: Na30 = nalidixic acid 30 μg/disc: Te30 =  tetracycline 30 μg/disc.

### Phenotype screening for ESBLs and AMPCs

The use of CDDST D68C and D73C (MAST™ Group Ltd, Liverpool, UK) kits enabled the confirmation of potential ESBL and AmpC β-lactamase producing DfSEC in this study. Of the 814 DfSEC isolates, 31 were phenotypically positive for the excretion of ESBL, 20 of the 31 isolates could additionally produce AmpC enzymes (Table 5). This result from the study is similar to the finding by Poulou et al. (2014) and Burgess et al. (2021) in similar comparative studies. The DfSEC isolate TL56S18 further showed the potential to produce *Klebsiella pneumoniae* carbapenem (KPC) hydrolysing enzymes, and PF55W18 also indicated the additional ability to produce metallobeta-lactamase (MBL) (Table 5). It is worthy to note that, KPC, OXA, and MBL are all carbapenem hydrolysing β-lactamases (Naas et al., 2005).

**Table 5:** DfSEC antimicrobial phenotype screening for ESBLs and AmpCs and their resistance to cefoxitin 30 μg/disc (FOX30).

		Differences in inhibition zones to D68C mm					Differences in inhibition zones to D73C* mm				FOX (30 mg) resistant	Resistant type
Farm	Isolate	B-A	D-C	D-B	C-A	D—C	B*-A*	C*-A*	D*-A*	E	≤ 10 mm	bla*ESBL*
Clearwaters (CW)	28-A18	7	0	1	8	0	2	6	6	21	+	ESBL+AmpC
	33-S18	5	2	2	5	2	0	4	5	20	+	ESBL+AmpC
	49-A18	3	5	6	4	5	0	6	5	22	+	ESBL+AmpC
Mill Road (MRD)	21-A18	14	4	4	14	4	4	15	17	22	+	ESBL+AmpC
	22-W18	6	0	0	6	0	2	2	2	17	+	ESBL+AmpC
	24-S17	4	5	7	4	5	1	1	3	21	+	ESBL+AmpC
	30-S17	6	0	1	7	0	3	2	2	21	–	ESBL
	33-S17	5	0	1	6	0	0	0	1	19	–	ESBL
	37-S17	5	0	0	5	0	0	1	1	25	–	ESBL
Peel Forest (PF)	17-S17	5	0	0	5	0	2	2	1	21	–	ESBL
	22-A18	5	2	3	6	2	0	0	0	25	–	ESBL
	24-A18	3	6	6	3	6	2	1	2	25	–	ESBL
	25-S18	6	5	4	5	5	1	4	2	20	+	ESBL+AmpC
	32-A18	5	6	5	4	6	3	2	1	21	+	ESBL+AmpC
	40-S17	5	0	1	6	0	0	1	0	22	–	ESBL
	45-S17	6	0	1	7	0	1	2	4	22	–	ESBL
	45-W18	3	1	1	3	1	4	6	4	24	+	ESBL+AmpC
	52-W18	6	0	1	7	0	5	4	4	25	+	ESBL+AmpC
	55-W18	7	0	1	8	0	2	2	2	8	+	ESBL+AmpC +OXA-48
	30-A18	3	2	5	6	2	0	2	2	21	+	ESBL
Peel Forest (PF)	14-A18	7	0	1	8	0	4	11	4	22	+	ESBL+AmpC
	15-A18	3	5	5	3	5	1	1	5	16	+	ESBL+AmpC
Totara Valley (TL)	12-A18	1	6	6	1	6	1	1	0	22	+	ESBL+AmpC
	1-S18	4	6	6	6	6	3	10	10	21	+	ESBL+AmpC
	23-A18	4	1	5	6	1	2	2	1	21	–	AmpC
	2-A18	4	5	5	1	5	0	2	2	24	+	ESBL+AmpC
	33-S18	5	0	1	6	0	4	2	4	20	–	ESBL
	54-S18	7	0	0	7	0	0	0	1	18	+	ESBL+AmpC
	56-S18	6	2	0	4	2	12	3	4	19	+	ESBL+KPC+AmpC
	87-A18	14	4	4	15	4	4	15	18	22	+	ESBL+AmpC
	11-A18	7	1	1	7	1	1	1	1	20	–	ESBL

Statistical analysis by binary logistic regression in Minitab19 indicated that there were no significant differences (*P* > 0.05) between the two farming systems with regards to the number of isolates positive for the excretion of β-lactam hydrolysing enzymes only. However, an odds ratio of 1.5 indicated more isolates from the conventional dairy farms released β-lactamase compared to isolates from the organic dairy farms.

The only isolate (PF55W18) out of 31 that was positive for the release of the OXA-48 hydrolysing enzyme was from the Peel Forest conventional dairy farm with the zone diameter of >10 mm to the temocillin+MBL inhibitor. The OXA type β-lactamases are poorly inhibited by clavulanic acid (Egorov et al., 2020; Naas and Nordmann, 1999) unlike ESBLs that are sensitive to clavulanic acid as an inhibitor (Musa et al., 2020; Stewart et al., 2020). Comparison between similar farming systems but different locations showed that the western located organic dairy farm of Totara Valley organic farm had eight isolates releasing ESBL, AmpC, or MBL hydrolysing enzymes with one isolate releasing resistant enzymes to multiple classes of antimicrobials while three isolates from the eastern located Clearwaters organic farm excreted only ESBL hydrolysing enzymes. According to Onishi et al. (1974) cephamycins, B and C are 50 to 170 times, respectively, more rapidly hydrolysed by β-lactamase produced by certain members of the *Enterobacteriaceae* family such as *Enterobacter cloacae* compared to the cephamycin cefoxitin due to cefoxitin being a poor substrate for the β-lactamase these organisms produce (Böhm et al., 2020).

### Phylogeny of DfSEC

The phylogenetic grouping of the 814 DfSEC isolates indicated that the B1 phylogenetic group predominated at 73.7%. The E phylogenetic group at 9.6% was the next most common, followed by group phylogenetic A at 5.8% and group C at 5.3%. The clade *I*+II and cladeIII+IV+*V* groups were 0.7% and 0.9% of the total, respectively. The B2 and D groups each represented 0.5% of the 814 isolates (Table 7). None of the DfSEC were assigned to the F group, and 3.1% of the isolates could not be placed in any of the presently recognised *E. coli* phylogenetic groups using the Clermont et al. (2013) protocol.

Statistical analysis showed no significant difference between the phylogenetic groups of the two farming systems of conventional and organic dairy farming, nor significant differences in the phylogenetic groupings between the farms (data not shown). Similarly, there were no significant differences between the phylogenetic groups according to the four sampling times by the Kurskal-Wallis ANOVA on ranks SigmaPlot 14.0 statistical analysis (data not shown).

The phylogeny group B1 has been found to be the dominant *E. coli* phylotype in the bovine environment (Fazel et al., 2019), as was reflected in our study. This group correspondingly showed a higher number of resistant isolates to the chosen antimicrobials compared to the other phylogeny groups (Table 6). The phylogeny group C, however, was third in dominance at 5.3% of the total number of isolates (*n* = 814) compared to the A and E phylogeny groups at 5.8% and 9.6%, respectively (Table 7). However, the phylogeny group C showed more resistance/intermediate resistance to tetracycline 30 μg, gentamicin 10 μg, and chloramphenicol 30 μg at four, four, and two compared to both phylogeny groups A at one, one and one and E at two, one and one, respectively. Also, 3.1% of the DfSEC isolates which were classified as unknown, had four isolates out of the total of 97 isolates that were resistance/intermediate resistance to cefoxitin 30 μg compared to the A and C groups at two and three isolates, respectively.

**Table 6:** . Number of DfSEC phylogeny groups showing resistance/intermediate resistance (RI) to selected antimicrobials.

Antimicrobials									
Phylogroups	Te30	Na30	CPD10	CN10	MEM10	CIP30	FOX30	C30	Total RI
A	1	1	1	1	1	0	2	1	8
B1	31	11	52	11	17	0	71	7	200
B2	0	0	0	0	0	0	0	0	0
C	4	0	1	4	0	0	3	2	12
D	0	0	0	0	0	0	0	0	0
E	2	0	5	1	1	0	10	1	20
F	0	0	0	0	0	0	0	0	0
cladeI+II	0	0	1	0	0	0	1	0	0
cladeIII+IV+*V*	0	0	0	0	0	0	0	0	0
Unknown	0	0	0	0	0	0	4	0	0
**Total RI**	38	12	66	17	19	0	97	10	260

**Table 7:** Phylogenetic groupings and distribution of DfSEC isolates (*n* = 814).

Phylogenetic group											
Farm-season-year	(n)	A	B1	B2	C	D	E	F	Clade I,II	Clade III-V	Unknown
CW-S-17	50	2	45	0	1	0	2	0	0	0	0
CW-A-18	50	1	40	0	6	0	2	0	0	0	1
CW-S-18	47	0	28	0	5	1	13	0	0	0	0
CW-W-18	47	4	26	0	2	1	13	0	0	1	0
TL-S-17	51	0	34	0	5	0	4	0	0	0	8
TL-A-18	47	3	36	0	0	0	0	0	1	0	7
TL-S-18	50	1	46	0	0	0	24	0	0	1	0
TL-W-18	47	7	35	0	0	0	5	0	1	1	3
MRD-S-17	68	4	54	2	3	0	4	0	0	0	1
MRD-A-18	55	4	42	1	0	1	6	0	1	1	0
MRD-S-18	50	2	38	1	3	0	6	0	0	0	2
MRD-W-18	50	9	32	0	0	0	9	0	0	0	3
PF-S-17	50	1	29	0	13	0	6	0	1	0	0
PF-A-18	55	0	38	0	3	1	9	0	2	2	0
PF-S-18	49	3	38	0	1	0	6	0	0	1	0
PF-W-18	48	6	39	0	1	0	2	0	0	0	0
Total	814	47	600	4	43	4	78	0	6	7	25
**%**		**5.8**	**73.7**	**0.5**	**5.3**	**0.5**	**9.6**	**0.0**	**0.7**	**0.9**	**3.1**

CW=Clearwaters organic dairy farm: TL = Totara valley organic dairy farm: MRD = Mill road conventional dairy farm: PF = Peel Forest conventional dairy farm: S-17 = spring of 2017: S-18 = spring of 2018: A-18 = autumn 2018: W-18 = winter of 2018.

## Discussion

### Conventional versus organic practices effect on DfSEC

The antimicrobial profile of the DfSEC isolates from the conventional dairy farms where a significant amount of antimicrobials herbicides, pesticides, and inorganic nitrogen-based fertilizers are frequently used indicated a higher percentage of resistant DfSEC isolates compared to isolates from the organic dairy farms during three of the four sampling-time points (Table 4). However, the organic dairy farms were not devoid of resistant strains, as the spring 2017 sampling indicated a significantly higher prevalence of resistant isolates from the organic system compared to the conventional. Since antimicrobials in the environment (especially the farm soil environment) are ubiquitous (Martínez et al., 2015; Peterson and Kaur, 2018; Tyc et al., 2017), it would be impossible to determine how much of it may be due to human activities, unless the quantity of antimicrobials from human activity deposited in that environment over a specified period were determined (Berendonk et al., 2015). This may explain the lack of significant differences by statistical analysis of the differences between resistances observed on organic farms compared to conventional farms in this study, but not the increased risks posed by usage on conventional farms as indicated by results in this study. In NZ, conventional dairy farming accounted for ca. 11% (10 230 kg of antimicrobials) of the national antimicrobial usage in 2017 (Ministry for Primary Industries, 2019) and this trend has been increasing (Ministry for Primary Industries, 2019). Since about 30–80% of antimicrobial used is excreted whole or as metabolites (Berendsen et al., 2015; Onyeanu et al., 2020; Sivagami et al., 2020), a significant quantum of antimicrobials are excreted onto conventional dairy farm soils and impact the soil microbiome's AMR status (Kampouris et al., 2021; Nadimpalli et al., 2020; Ojemaye et al., 2020), as opposed to organic dairy farming. For instance, a 2014–15 study in five different regions in New Zealand including North Canterbury indicated **∼**4.8 mg of active ingredient/population correction unit (PCU, defined as the mass of active ingredient divided by total biomass) to ∼684 000 cows (Al-Ahmad et al., 2001). This is substantiated by other authors, whereby the use of antimicrobials and other agrochemicals in conventional dairy and other agricultural husbandry systems increases the amount of AMR bacteria and antimicrobial-resistant genes (ARGs) in the bacteria compared to the limited/non-usage of these chemicals on organic farms (Adebowale et al., 2019; Awad et al., 2014; Nhung et al., 2016; Österberg et al., 2016; Pollock et al., 2020; Schwaiger et al., 2010). Mastitis is the most concerning pathology on a dairy farm (Ruegg, 2017; Ruegg and Petersson-Wolfe, 2018; Ruegg and Reinemann, 2002). In New Zealand, about 14/100 cows/annum of the milking herd on a bovine dairy farm would be affected by mastitis (McDougall and Compton, 2002). The best treatment of mastitis is the use of antimicrobials because the main causative agents are bacteria including *E. coli* (Bianchi et al., 2019; Kibebew, 2017; McDougall, 2002) and AMR *E. coli* was found in dairy farm paddock feaces in a New Zealand study (Burgess et al., 2021). In most OECD countries including New Zealand, milk from cows with mastitis being treated with antimicrobials must be disposed of, until the withholding period of the drug is over (Anika et al., 2019). The milk is either fed to calves on the farm or disposed of in the sewage, later to be used for irrigating the fields (Lago et al., 2011; Ruegg, 2017; Ruegg and Reinemann, 2002). The mastitis-causing bacteria from such milk may thus end up in the soils of the fields, through the digestive system of the calves and the sewage used for irrigation (Burgess et al., 2021; Houlbrooke et al., 2004). Polacek (2015) explained that pathogenic *E. coli* strains possess special features like *curli* fimbriae for adhesion, invasion of host cells and to protect themselves with biofilm formation to enable them to persist in the mammalian system to avoid destruction by antimicrobials.

### The phylogenetic grouping of DfSEC

In this study, the *E. coli* phylogeny group B1 was the predominant group (73.7%) in the 814 DfSE isolates collected from the dairy farms. This was similar to other studies that have looked at the phylogenetic grouping of *E. coli* isolates of bovine origin (Blum and Leitner, 2013; Milanov et al., 2015; Suojala et al., 2011). The phenomenon of different members of the various *E. coli* phylogenetic groups dominating in prevalence amongst a particular species of animals and humans as well as niches has been demonstrated in numerous studies (Karami et al., 2020; NandaKafle et al., 2021; Nowrouzian et al., 2005). Even for a particular animal species, the distribution of the various phylogenetic groups as commensals or pathogens may belong to different phylogenetic groups (Jang et al., 2017; Keane, 2016; Mercat et al., 2016; Suojala et al., 2011). Some studies that have looked at *E. coli* from the bovine environment such as soils and manure have indicated the phylogeny group B1 to be most predominant (Blum and Leitner, 2013; van Overbeek et al., 2020). This is similar to results in this study with the B1 group dominating at 74% of the DfSEC isolates. In this study, the second most common of the *E. coli* phylogeny groups was type E at 9.6% using the Clermont et al. (2013) method. This method can tease out *E. coli* isolates that were previously grouped into group *D* + *E* into either D, E, or clade II+III+IV and A + C into either A or C groups as opposed to a previous phylogeny typing method that other authors have used (Gordon, 2010). Authors who have used the less sensitive Clermont et al. (2000) method have indicated the phylogeny group D to be next in common following groups B1 and A in pathological cases of mastitis (Suojala et al., 2011; Zhang et al., 2018) and metritis (Gonzalez Moreno et al., 2020; Silva et al., 2009). Similarly, while the phylogenetic group A had featured in most studies, group C had little mention but, in this study, 5.3% of the DfSEC belonged to the C group. This may be because, in this study, DfSEC isolates that would have been typed as A or A + C were re-typed with the trp*Agp*C primers to differentiate A + C into As and Cs. Other studies had used the earlier version, Clermont et al. (2000), typing protocol and had not been able to differentiate some *E. coli* isolates into their phylogenetic groups as robustly as provided by Clermont et al. (2013). This was shown by Logue et al. (2017) in a study.

### Possible origins of DfSEC

*E. coli* is of gastrointestinal origin (rumenoinstinal in bovine), but there is evidence to suggest strains associated with mastitis may have genotype sequences not shared by commensal strains (Jung et al., 2021). In this study, it may be argued that the members of the phylogeny group A, may have originated from the mammary glands of the cows with mastitis or secretions and aborted foeti from cows with endometritis (Haimerl et al., 2018; Wagener et al., 2017) and metritis (de Boer, Heuer, Hussein, and McDougall, 2015; Piersanti et al., 2019). A New Zealand study of pathogens in raw milk collected monthly for a year from five major bovine dairy regions found that *E. coli* was present at <100 cfu/ml in 99% of samples and exceeded 10^3^ cfu/ml in 0.7% of samples (Hill et al., 2012). Mastitis is a common pathology in dairy cows globally (Blum, Heller, Jacoby, Krifucks, & Leitner, 2017), and in New Zealand (Hill et al., 2012; Petrovski et al., 2009). According to Zhang et al. (2018) and de Cassia Bicudo and Oba (2019), the *E. coli* phylogenetic group A is most commonly associated with mastitis and metritis, respectively. Of the 48 strains recovered in our study, just five showed resistance to at least one antibiotic and of these, only one was considered to fit the ESBL phenotype (Table S1)

In our study, the predominant phylogeny group B1 also contained the largest number of resistant isolates, echoing other studies (Fazel et al., 2019; Silva et al., 2020). Also in this study, the use of the Clermont et al. (2015) protocol enabled the dissociation of the phylogeny groups A + C and the *D* + E phylogeny groups and their antibiotic resistance/intermediate resistance was able to be determined. The phylogeny group E showed the next in level of resistance to the second-generation cephalosporin, cefoxitin 30 μg, but against tetracycline 30 μg, phylogeny group C was next highest in frequency to the dominant B1 phylogeny group although most studies indicated phylogeny groups A and D as next in dominance to B1 in commensal *E. coli* from the bovine environment (Arimizu et al., 2019). The differences in results may be differences in the phylogenetic typing methods, as the other authors used the ClemonType protocol which employs a web-based interface and allows a given strain to be assigned to *E. albertii, E. fergusonii, Escherichia* clades I–V, E. coli sensu stricto as well as to as the seven phylogeny groups described here (Beghain et al., 2018).

Only 0.5% of our 814 DfSEC isolates were assigned to phylogenetic group B2. This group is rarely associated with cattle (Liu et al., 2014; Madec et al., 2012) but it is the predominant group associated with humans and their companion animals of cats and dogs (Bogema et al., 2020; Carlos et al., 2010, Collis et al., 2019; Kidsley et al., 2020; Toombs-Ruane et al., 2017; Harada et al., 2012; Mateus et al., 2013; Zogg et al., 2018). While such domestic pets are common on New Zealand dairy farms, their interaction with the grazing environment is likely intermittent at best, potentially limiting their exposure and thus opportunity for acquisition.

## Conclusion

Antimicrobial resistance is a global problem that is best tackled with combined information from the different regions of the world. Data on the possible origins, prevalence, and mode of spread from any region of the globe is relevant to mitigation approaches to the problem. This is more so because the migration of people, wildlife, movement and currents of wind, and water bodies may cause the spread of resistant organisms and the ARGs they may carry to different parts of the globe (Hooban et al., 2020; Moore et al., 2020).

This study provided evidence that continual and increased use of antimicrobials and other agrochemicals in the New Zealand dairy industry may increase the prevalence and possibly, the spread of antimicrobial-resistant bacteria from their soils to other environments. The study also highlights the possibility of human-sourced infectious *E. coli* resistant to some antimicrobials getting into the cattle environment of a dairy farm soil. This is explained by *E. coli* of phylogenetic A and B2 being most commonly associated with humans but rarely with bovine being found in soils closely associated with bovine, humans, cats, and dogs as is common on NZ dairy farms. The reverse has been shown by other authors as Shiga toxin-producing *E. coli* (STEC) O157:H7 and other bacterial infections in humans have originated from cattle environment (Gilpin et al., 2008; Gilpin et al., 2020; Jaros et al., 2013).

This study may have indicated husbandry practices in the dairy farming industry that possibly contribute to the prevalence and spread of antimicrobial resistance microbes in their environment. As pathogenic *E. coli* resistant to certain antimicrobials from cows with mastitis, endometritis, and or metritis may be found in the dairy farm soil, such bacteria may spread resistance genes horizontally and vertically to other microbes and may eventually infect humans and other mammals associated with dairy farms (Jechalke et al., 2014; Li et al., 2019). This may be attributed to the husbandry practice of disposing of milk from cows with mastitis into the farm sewage and later used for irrigation. Secondly, the feeding of calves with milk from cows with clinical and or subclinical mastitis, knowingly or unknowingly, respectively, (Cunha et al., 2021; Oliver et al., 2020) may lead to the spread of these organisms to other body systems and through the faeces, into the soil and subsequently to humans and other mammals. Additional studies are required to determine if our observations made in the Canterbury region are widely applicable in farming landscapes across New Zealand and indeed beyond; nonetheless, the relevance of our findings to increasing AMR rates is self-evident.

## Declaration of Competing Interest

The authors declare the following financial interests/personal relationships which may be considered as potential competing interests: Stephen L. W. On reports financial support was provided by NZ Food Safety and Science Research Centre.

## Appendix Supplementary materials

Image, application 1
